# Prediction of Maximal Oxygen Consumption in Cycle Ergometry in Competitive Cyclists

**DOI:** 10.3390/life13010160

**Published:** 2023-01-05

**Authors:** Iva Jurov, Janez Toplišek, Marta Cvijić

**Affiliations:** 1 Clinical Institute of Occupational, Traffic and Sports Medicine, University Medical Centre Ljubljana, 1000 Ljubljana, Slovenia; 2 Department of Cardiology, University Medical Centre Ljubljana, 1000 Ljubljana, Slovenia; 3 Faculty of Medicine, University of Ljubljana, 1000 Ljubljana, Slovenia

**Keywords:** oxygen consumption, aerobic exercise, physiology, physical performance, sports medicine

## Abstract

Models for predicting maximal oxygen consumption (VO_2max_) in average adults might not be suitable for athletes, especially for competitive cyclists who can have significantly higher VO_2max_ than normally active people. The aim of this study was to develop a clinically applicable equation for predicting VO_2max_ during cycle ergometry in competitive cyclists and to compare its accuracy to the traditional American College of Sports Medicine (ACSM) equation. Maximal cycle ergometry tests were performed in 496 male and 84 female competitive cyclists. Six predictors were initially used to model the prediction equation (power output, body weight, body height, fat mass, fat-free mass and age). Power output and body weight were the most important parameters in the model predicting VO_2max_. Three new equations were derived: for male (VO_2max_ = 0.10 × PO − 0.60 × BW + 64.21), female cyclists (0.13 × PO − 0.83 × BW + 64.02) and the non-gender-specific formula (0.12 × PO − 0.65 × BW + 59.78). The ACSM underestimated VO_2max_ in men by 7.32 mL/min/kg (11.54%), in women by 8.24 mL/min/kg (15.04%) and in all participants by 7.45 mL/min/kg (11.99%), compared to the new equation that underestimated VO_2max_ in men by 0.12 mL/min/kg (0.19%) and in all participants by 0.65 mL/min/kg (1.04%). In female cyclists, the new equation had no relative bias. We recommend that medicine and sports practitioners adapt our proposed equations when working with competitive cyclists. Our findings demonstrate the need to evaluate prediction models for other athletes with a special focus on disciplines that demand high aerobic capacity.

## 1. Introduction

In sports physiology, the cardiopulmonary exercise test is utilized for testing exercise capacity, for determining training plans and looking for causes of exercise intolerance. It is also used for monitoring effects of interventions and, in normally active individuals, it provides important information in patient diagnosis and management [1]. Indirect calorimetry in exercise testing is considered as a gold standard to detect maximal oxygen uptake (VO_2max_), which is a primary indicator of cardiorespiratory fitness during the incremental test. Measuring VO_2max_ needs to be performed by a skilled technician by using standardized exercise treadmill protocols or cycle ergometry [2]. Indirect calorimetry with a gas analyser is required to determine VO_2max_. Measured VO_2max_ is usually compared to predicted values in order to estimate an individual’s suboptimal values. In addition, VO_2max_ also has to be estimated when direct measurement is not feasible [3]. Predicting VO_2max_ can be carried out with several prediction models [1]. Traditionally, the American College of Sports Medicine (ACSM) equation is used, which was determined 40 years ago. In recent years, its widespread use has been questioned by some researchers aiming to optimize its estimation in specific populations.

Endurance athletes, especially competitive and professional, are also a specific population that significantly differs from normally active individuals in their high aerobic capacity. During the incremental test, athletes, in general, achieve higher VO_2max_ values than their non-athletic counterparts, but trained endurance athletes exhibit the highest values of all sport disciplines. This is also the case in competitive cyclists. Faria et al. concluded that VO_2max_ values in successful riders are as high as 74 mL/kg/min [4]. Although a high VO_2max_ is a prerequisite in professional cyclists, it is not the only factor influencing results. In moderately trained cyclists, other tests, such as functional threshold power, are used and can predict performance as well [5].

When selecting reference values for VO_2max_, it is important that the individual tested matches the population in which the reference values were obtained [1]. Traditional equations for predicting VO_2max_ in cardiopulmonary exercise testing, such as the ACSM equation [6], are based on the non-elite, normally active population [3]. Although they are used for athletes as well, they might not be the most appropriate for predicting VO_2max_ in competitive cyclists. Similar conclusions were found in other specific populations, such as in patients with heart failure [7], coronary artery disease patients [8] and also in healthy adults [9,10], where new and improved prediction models have been presented and show greater accuracy.

In cyclists, cycle ergometry is the most suitable type of incremental testing in the laboratory. To achieve absolutely maximal values, it is advised to use the cyclist’s own bike that is attached to the ergometry system. In this way, the problem of the cyclists not being able to configure the ergometer to their normal riding posture is eliminated [4]. The test is terminated based on different termination criteria: an athlete’s volitional fatigue, pedal cadence cannot be maintained, a given workload cannot be maintained, a plateau in VO_2_ or a difference in metabolic parameters (a rise in respiratory exchange ratio above 1.0, 90% of predicted heart rate). One or more criteria can be used, but a plateau in VO_2_ is generally considered as the most important marker of reaching VO_2max._ Incremental tests in competitive cyclists are usually conducted on a regular basis to evaluate their health and performance. Depressed VO_2max_ may not only be a sign of illness but also of fatigue or overtraining. If cyclists are fatigued, power output during the incremental test is typically also affected [11]. Finally, VO_2max_ can be used for training monitoring during training modifications and adaptations [12,13]

Comparing measured values of VO_2max_ of an athlete to predicted VO_2max_ values of normally active people could consequently lead to misdiagnosis. This is why developing a VO_2max_ equation specific for endurance athletes with typically high VO_2max_ is needed. The aim of this research was to develop a clinically applicable equation for predicting VO_2max_ during cycle ergometry in competitive cyclists and to compare its accuracy to the traditional ACSM equation based on a larger sample of 580 competitive cyclists.

## 2. Materials and Methods

Healthy competitive road cyclists with a valid license were included in this study as part of an annual cycling performance analysis that is set before the competitive period in the season. One day prior to the test, they refrained from high-intensity exercise. They were well rested and received instructions on how to be well hydrated. Informed consent was obtained before starting the procedures.

Two experienced physiologists performed all procedures in the same exercise laboratory in 5 consecutive years (2014–2019). Results of the test were retrospectively analysed for the purpose of this research. This study conformed to the standards set by the latest version of the Declaration of Helsinki. Informed consent was obtained for experimentation with human subjects. Institutional ethical committee approved this study (0120-202/2020/5).

After a 15 min warm-up, a graded exercise test was performed using cycle ergometry (Cyclus 2, Leipzig, Germany). Ventilatory and gas data (measured with V2 mask, Hans Rudolph, Shawnee, KS, USA of appropriate size) were collected during the incremental test with indirect calorimetry (metabolic cart K5, Cosmed, Albano Laziale, Italy). The workload was constantly increased until volitional exhaustion. Only maximal incremental tests were included in the analysis, defined as respiratory exchange ratio R ≥ 1.0, a reached plateau in VO_2max_, BORG scale ≥ 8. The test was also terminated if an athlete could not maintain cycling cadence above 60. Two modified Conconi protocols, commonly used in elite athletes [14], were used based on cyclists’ age and body mass. Cyclists under 17 years of age or weighing less than 50 kg started protocol at 60 Watts and increased 15 Watts every minute (the 60 + 15 W protocol) and cyclists above 17 years of age and weighing more than 50 kg started protocol at 100 Watts and increased 20 Watts every minute (the 100 + 20 W protocol). Using breath-by-breath data, the VO_2max_ was determined as the average of the 5 s highest values during the last 30 s in the incremental test. Ambient temperature during all procedures was 21 °C. Metabolic cart was calibrated prior to each of the measurements.

Prior to body composition measurement, participants received instructions on how to be adequately hydrated. Body composition was assessed with bioelectrical impedance (Biospace Inbody 720, Cerritos, CA, USA).

Descriptive statistics (average ± standard deviation) was used for representing measured and predicted values of VO_2max_ and for all baseline characteristics. Pearson correlation coefficients were used to examine the relationship between predictors before performing multiple regression analysis. The generalization of the model was tested by splitting the data randomly on 70% and 30% of participants in each of the groups (male, female and non-gender specific). The model was derived from data of 70% and was confirmed on the remaining 30% by forcing the model [15]. The model is accurate for the sample and generalizable for the population. Assumptions for multiple regression (non-zero variance, multicollinearity, homoscedasticity, normality of distribution, independence, linearity) were checked. Quantitative variable types were used in multiple regression analysis. The predictors did not have variances of 0. We excluded parameters that had correlation above 0.8 between them to confirm the assumption of no multicollinearity. Variance inflation factor (VIF) (well below 10) and tolerance statistics (well above 0.2) were also checked.

**Figure A1:**
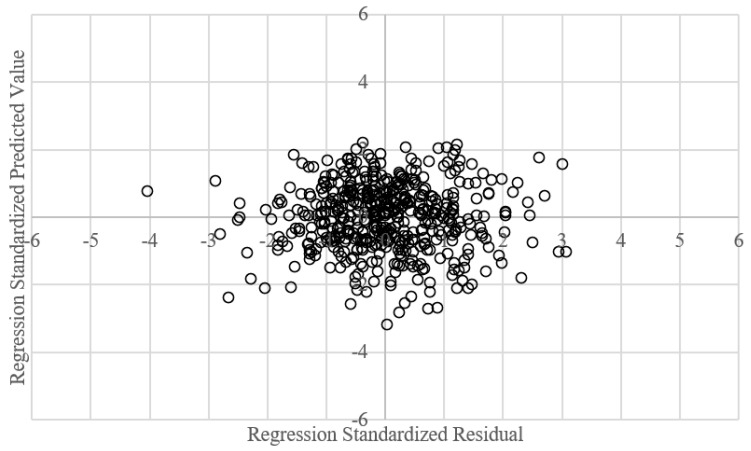
The plot of standardized residuals against standardized predicted values.

**Figure A2:**
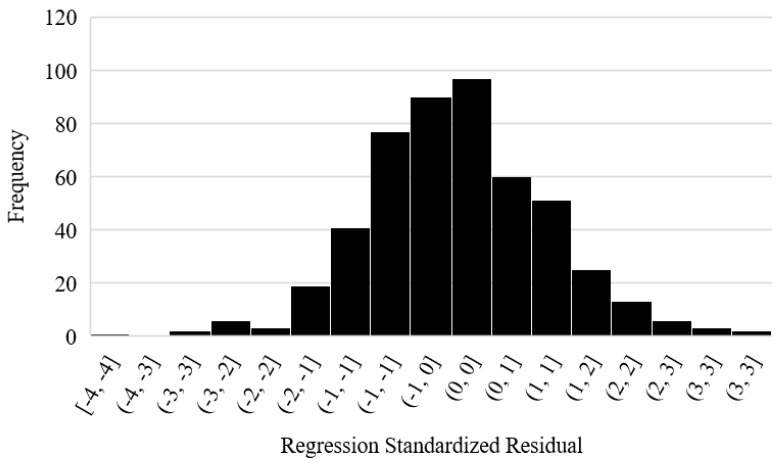

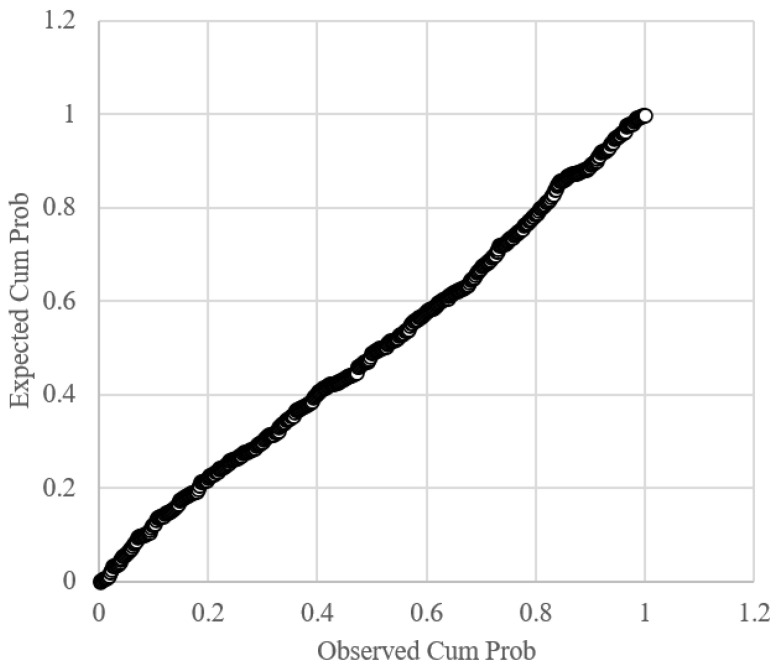
Normality of residuals presented with a histogram and normal probability plot of the residuals.

Durbin–Watson test for independent errors was 1.657, 1.854 and 1.536 for males, females and the non-gender-specific group, respectively. As seen in Figure A1 (Appendix A), the points are randomly and evenly dispersed throughout the plot, which means that assumptions of linearity and homoscedasticity were met. To test the normality of residuals, we present the histogram and normal P-P plot of regression-standardized residual (Figure A2, Appendix A).

**Figure 1:**
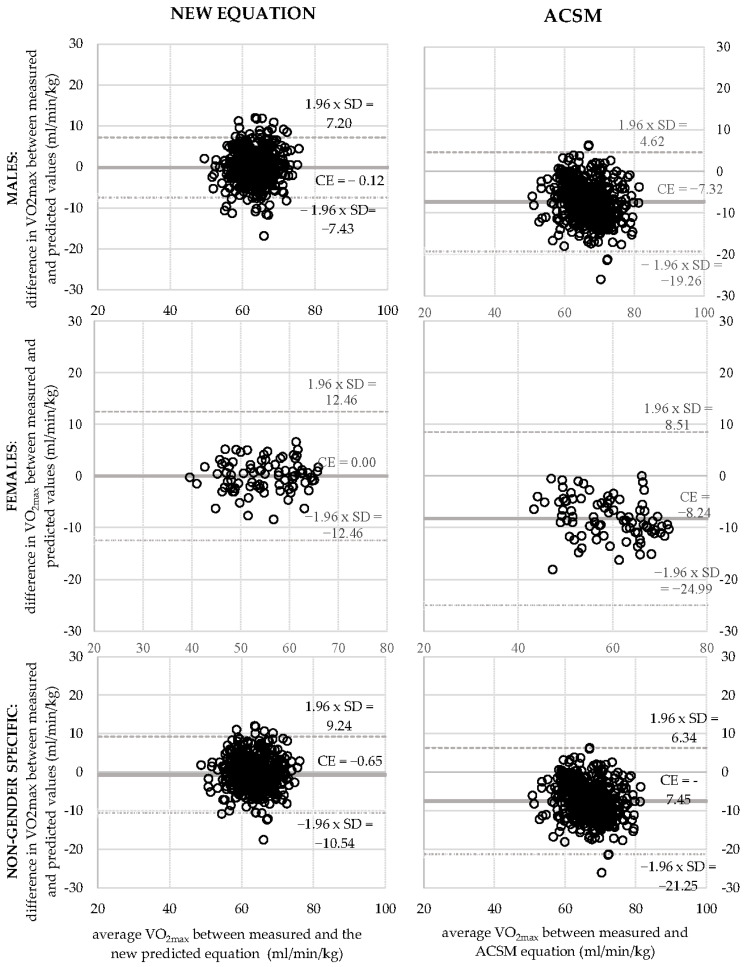
Bland–Altman plots comparing measured VO_2max_ and predicted VO_2max_ using the new gender—specific equations and the American College of Sports Medicine (ACSM) equation.

Bland–Altman plots were constructed to graphically illustrate the variance between measured VO_2max_ and VO_2max_ generated from the new and the ACSM equations (Figure 1. Constant error (bias) and standard error of the estimate (SEE) were calculated.

## 3. Results

The baseline characteristics of participants are presented in Appendix B (Table A1). Cyclist were recruited from 21 competitive female and male road-cycling teams. On average, male cyclists were younger than female cyclists, they were higher and heavier, they had less body fat and a greater fat-free mass. Their maximal power output was greater, both when measured absolutely (W) and relatively (W/kg). The VO_2max_ in males was greater than in females.

The stepwise method was used in the multiple-regression analysis to indicate which predictors are the best for predicting the measured VO_2max_. Multiple regression was performed on males, females and on the entire sample to develop the gender non-specific equation. Initially, six predictors were considered based on previously used variables [1]: power output (W), body weight (kg), body height (cm), skeletal muscle mass (kg), fat mas (%) and age.

**Figure 2:**
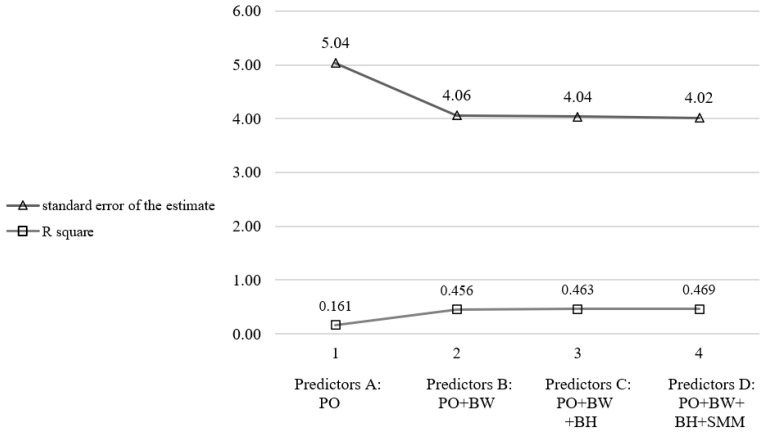
Average standard error of the estimate with variables considered in the multiple regression models (predictors in the models A–D) for male cyclists. Lower values of standard error of the estimate and higher of R square indicate greater predictive value of the model (PO = power output, BW = body weight, BH = body height, SMM = skeletal muscle mass).

To test the six predictors, at least 60 participants were required for the analysis [15]. Since skeletal muscle mass and fat mass were highly correlated, fat mass was excluded from further analysis. Age predictor was also excluded since it over- and underestimated predicted VO_2max_ based on confidence intervals. The stepwise technique was repeated with the remaining four predictors. Considering average squared error (average standard error of the estimate) and R square as “goodness-of-fit parameters”, model 2 was the most appropriate since adding variables in models 3 and 4 did not change the model significantly (Figure 2).

In Appendix B (Table A2), we present the influence of parameters on the measured VO_2max_. Results revealed that the power output has the biggest influence on VO_2max_ followed by body weight. However, the other two predictors (body height and skeletal muscle mass) have a lower impact on the outcome parameter (Table A2).

The model using power output and body weight was chosen in all three groups based on having a high R square (other models with additional parameters do not increase R square appreciably). In Table 1, we present gender-specific and non-gender-specific formulas. R square was 0.456 in male, 0.671 in female cyclists and 0.589 in the non-gender-specific group.

A comparison of relative bias in the new equations and ACSM equation is presented in Figure 1. ANOVA was also performed to compare the ACSM equation and the new equation (Appendix B, Table A3). The ACSM underestimated VO_2max_ in men by 7.32 mL/min/kg (11.54%), in women by 8.24 mL/min/kg (15.04%) and in all participants by 7.45 mL/min/kg (11.99%), compared to the new equation that underestimated VO_2max_ in men by 0.12 mL/min/kg (0.19%) and in all participants by 0.65 mL/min/kg (1.04%). In female cyclists, the new equation had no relative bias (0.00%).

## 4. Discussion

The aim of this study was to develop a clinically applicable equation for predicting VO_2max_ during cycle ergometry in competitive cyclists and to compare its accuracy to the traditional ACSM equation. Based on a large database of competitive cyclists, we present our new equations (gender-specific and -non-specific), which proved to be more accurate for competitive cyclists that the traditional ACSM equation.

The new prediction models require the same parameters as the ACSM equation [6] (power output and body weight of the participant) but are improved based on a big database of cyclists included. Similarly to the ACSM, they are simple to use. Based on the results, we would suggest using gender-specific formulas, since the number of both female and male cyclists included in this research was high. The gender-non-specific formula was derived to present the differences if gender is not considered. In addition, the gender-non-specific formula can be used in cases where software programs do not enable usage of two (but only one) equations for predicting VO_2max_ before initiating the incremental test. In this case and as supported by this paper, it is better to use the gender-non-specific formula than the ACSM equation.

Average VO_2max_ 62.2 mL/min/kg confirmed that cyclists included in this study have superior physical fitness based on Cooper Institute for Aerobics Research to classify the degree of fitness compared to normally active individuals [16]. Based on their average maximal power output (5.90 W/kg in male and 5.19 in female cyclists), they are considered top-level competitive cyclists [17]. This supports the prediction that an equation that is based on normally active individuals (the ACSM) might not be appropriate for competitive cyclists.

The applicability of the ACSM equation was tested in specific populations in recent years. Commonly used equations were improved by newly derived equations on the normally active general population [9] and specific populations, such as coronary artery disease patients [8] and heart-failure patients [7]. The same was carried out in this paper as well. Our results suggest that the previously used ACSM equation might not be the most suitable in competitive cyclists. In our sample, the ACSM equation was less accurate than our new prediction model. The ACSM underestimated VO_2max_ in men by 11.54%, in women by 15.04% and in all participants by 11.99%. Our equation had a smaller relative bias. On average, it underestimated VO_2max_ in men by only 0.19% and in all participants by 1.04%. In female cyclists, it had no relative bias (0.00%). Since ACSM is based on a different population aged 20 years or more [3], the findings are not surprising. ACSM is also not derived from competitive cyclists.

The results of this study are in line with research by Koutilanos et al. [18] on male competitive athletes. They concluded that ACSM’s equation is not capable of accurately predicting VO_2max_ in athletes aged 18–37 years. In contrast to Koutilanos et al., our study includes female athletes as well. Since female cyclists are much less studied than their male counterparts [19], we believe this is an important aspect of this research. The ACSM equation is certainly useful in adult persons that are normally active. However, based on these findings, its use might be limited in competitive cyclists. Comparing measured values of cyclists during cycle ergometry to more accurately predicted values can lead to better disease diagnosis, disease management, better training optimization, et cetera. If values are compared to the ACSM-predicted values, suboptimal VO_2max_ can easily be unrecognized. In addition, female athletes do not exhibit the same VO_2max_ values as male athletes and using specific formulas is vital for better athlete management in a sport medicine setting.

The new equation needs only two predictors and is easy to use when performing the incremental test without indirect calorimetry. Malek et al. [20] cross-validated VO_2max_ prediction equations on samples of aerobically trained males and females. They concluded that the equation by Storer et al. was the most accurate. Like our model, this equation calculated VO_2max_ by using power output, body weight, but it also requires age as a predictor. Since it was designed based on 115 males and 116 females, aged 20–70, from the general population, it is not surprising that age had an important impact on the outcome [21]. In our sample, however, the sample was more homogenous (age 17.6 ± 3.5). The average age of our sample is also lower since most competitive cyclists included in this research were tested at the time when they just started competing not only nationally, but also at the international level. In addition, sample size in our research was much larger (580 participants compared to 231). Only accurate predicted VO_2max_ values are useful in the clinical setting when dealing with competitive athletes. Therefore, we recommend that medicine and sports practitioners adapt our proposed equation when working with competitive cyclists.

### Limitations

Some limitations of this study should be noted. Cyclists in this study are Caucasian so suggested prediction models might not be attributed to other ethnic groups. More research is needed to confirm if any differences exist. When we calculated the gender-non-specific equation, the group included far more male than female cyclists. To determine a more accurate prediction model, the number of subjects should be more balanced. The reason for less women in our sample is that female competitive cyclists are less common than male cyclists. We assume that the gender-non-specific equation might be different if genders were equally represented in the multiple-regression analysis. Still, we believe the new prediction equation could be more accurate than the ACSM equation. Finally, average age in the male group is lower than in the female group. This is a result of the fact that more “younger” cyclists come to exercise physiology centres where measurements for this article were performed, and some “older” cyclists train and get tested abroad with their pro-cycling teams. The number of young male cyclists is also much higher than female cyclists in the country of measurement and this resulted in slightly different age groups. Nevertheless, since the participants in our paper compete nationally and internationally, we believe they are a good representation of competitive cyclists. Additional studies are needed to explore whether this equation could be used on other specific groups, such as off-road cyclists, mountain bikers or in triathletes.

## 5. Conclusions

We present two gender-specific equations and a non-gender-specific equation that are based on a large database of competitive cyclists. The current results demonstrate that new prediction equations are more accurate for predicting VO_2max_ in competitive cyclists than the traditional ACSM equation. The equations require only power output and body-weight values, which makes them easy to use. The new equations are more accurate, they have lower bias and have a very low standard error in the estimate. They can be used in a setting where indirect calorimetry to measure VO_2max_ is not possible and to evaluate measured values to predicted values for evaluation. This can serve in the clinical setting for disease detection, disease management and also in the monitoring of performance and training adaptations. Finally, based on the results of this study, the traditionally used ACSM is not suitable for competitive cyclists based on great underestimation, both in male and female cyclists.

Future studies are needed to verify if the proposed prediction model could also be suitable for other disciplines with similar physiological demands, such as triathlon or mountain-bike cycling. There is also a need to develop new prediction models for disciplines with different physiological characteristics. This might be especially useful for cardiovascular preparticipation screening examination in disciplines where cardiovascular risk is increased.

## Figures and Tables

**Table 1:** New prediction equations for VO_2max_ in competitive cyclists.

	VO_2max_ (mL/min/kg)
**male cyclists**	0.10×PO−0.60×BW+64.21
**female cyclists**	0.13×PO−0.83×BW+64.02
**gender non-specific**	0.12×PO−0.65×BW+59.78

VO_2max_ = maximal oxygen uptake, PO = maximal power output in Watts, BW = body weight in kg.

## Data Availability

The datasets used and analysed during the current study are available from the corresponding author on reasonable request.

## Abbreviations

VO_2max_	Maximal oxygen uptake
ACSM	American College of Sports Medicine
SEE	Standard error of the estimate
PO	Maximal power output
BW	Body weight
BH	Body height
SMM	Skeletal muscle mass

## Appendix B

**Table A1:** Baseline characteristics of cyclists.

	Males (n = 496) Mean + SD		Females (n = 84) Mean + SD	
**age (years)**	**17.14**	2.72	20.18	5.59
**height (cm)**	178.67	6.73	166.04	5.57
**body mass (kg)**	66.72	7.58	57.45	6.46
**fat mass (%)**	9.29	3.87	17.34	4.67
**fat free mass (%)**	51.43	1.68	45.98	2.8
**maximal power output (W)**	393.74	56.48	296.05	45.05
**maximal power output (W/kg)**	5.90	0.56	5.19	0.79
**maximal oxygen consumption (ml/min/kg)**	63.43	5.49	54.82	7.02

**Table A2:** Parameters used in the stepwise multiple-regression analysis and their influence on the VO_2max_ in male cyclists.

	Unstandardized Coefficients	Standardized Coefficients			95% Confidence Interval for B	
	Std. Error	Beta	t	Sig.	Lower	Upper
**constant**	7.186		9.874	0.000	56.840	85.078
**PO**	0.005	1.023	20.087	0.000	0.090	0.109
**BW**	0.047	−0.698	−10.837	0.000	−0.598	−0.415
**BH**	0.048	−0.184	−3.151	0.002	−0.244	−0.057
**SMM**	11.628	0.084	2.324	0.021	4.178	49.872

(VO_2max_ = maximal oxygen uptake, PO = maximal power output, BW = body weight, BH = body height, SMM = skeletal muscle mass).

**Table A3:** ANOVA testing effect of different equations used—for all groups (male, female and gender-non-specific) there are statistically significant differences between the new equation and the ACSM aquation.

		Sum of Squares	df	Mean Square	F	Sig.
**male cyclists**	new equation	4657.287	190	24.512	3.339	0.000
	ACSM	12,573.567	190	66.177	3.479	0.000
**female cyclists**	new equation	3319.783	72	46.108	14.312	0.000
	ACSM	6022.488	72	83.646	23.317	0.000
**gender non-specific**	new equation	11,173.397	221	50.558	5.071	0.000
	ACSM	22,144.828	221	100.203	5.491	0.000

## References

[1] Palange P., Laveneziana P., Neder J.A., Ward S.A. (2018). Clinical Exercise Testing.

[2] Balady G.J., Arena R., Sietsema K., Myers J., Coke L., Fletcher G.F., Forman D., Franklin B., Guazzi M., Gulati M. (2010). Clinician’s Guide to Cardiopulmonary Exercise Testing in Adults: A Scientific Statement from the American Heart Association. Circulation.

[3] Thompson W.R., Gordon N.F., Pescatello L.S., American College of Sports Medicine (2010). ACSM’s Guidelines for Exercise Testing and Prescription.

[4] Faria E.W., Parker D.L., Faria I.E. (2005). The Science of Cycling. Sport. Med..

[5] Sørensen A., Aune T.K., Rangul V., Dalen T. (2019). The Validity of Functional Threshold Power and Maximal Oxygen Uptake for Cycling Performance in Moderately Trained Cyclists. Sports.

[6] Liguori G., Feito Y., Fountaine C., Charles J., Roy B., American College of Sports Medicine (2021). ACSM’s Guidelines for Exercise Testing and Prescription.

[7] Kokkinos P., Kaminsky L.A., Arena R., Zhang J., Franklin B., Kraus W., Triantafyllidi H., Benas D., Whellan D.J., Myers J. (2020). New Equations for Predicting Maximum Oxygen Uptake in Patients with Heart Failure. Am. J. Cardiol..

[8] Jang W.Y., Kang D.O., Park Y., Lee J., Kim W.-S.W., Choi J.Y., Roh S.-Y., Jang Y., Park S.-H., Kim W.-S.W. (2020). Validation of FRIEND and ACSM Equations for Cardiorespiratory Fitness: Comparison to Direct Measurement in CAD Patients. J. Clin. Med..

[9] Myers J., Kaminsky L.A., Lima R., Christle J.W., Ashley E., Arena R. (2017). A Reference Equation for Normal Standards for VO2 Max: Analysis from the Fitness Registry and the Importance of Exercise National Database (FRIEND Registry). Prog. Cardiovasc. Dis..

[10] Kokkinos P., Kaminsky L.A., Arena R., Zhang J., Myers J. (2018). A New Generalized Cycle Ergometry Equation for Predicting Maximal Oxygen Uptake: The Fitness Registry and the Importance of Exercise National Database (FRIEND). Eur. J. Prev. Cardiol..

[11] Spragg J., Leo P., Swart J. (2022). The Relationship between Training Characteristics and Durability in Professional Cyclists across a Competitive Season. Eur. J. Sport Sci..

[12] Duc S., Urianstad T., Rønnestad B.R. (2022). Adding Vibration During Varied-Intensity Work Intervals Increases Time Spent Near Maximal Oxygen Uptake in Well-Trained Cyclists. Int. J. Sports Physiol. Perform..

[13] Hebisz R., Hebisz P., Danek N., Michalik K., Zatoń M. (2022). Predicting Changes in Maximal Oxygen Uptake in Response to Polarized Training (Sprint Interval Training, High-Intensity Interval Training, and Endurance Training) in Mountain Bike Cyclists. J. Strength Cond Res..

[14] Lucia A., Hoyos J., Chicharro J.L. (2001). Physiology of Professional Road Cycling. Sport. Med..

[15] Field A.P. (2018). Discovering Statistics Using IBM SPSS Statistics.

[16] Cooper Institute (2017). Principles of Health and Fitness for Fitness Professionals.

[17] Faria E.W., Parker D.L., Faria I.E. (2005). The Science of Cycling: Factors Affecting Performance—Part 2. Sports.

[18] Koutlianos N., Dimitros E., Metaxas T., Deligiannis A.S., Kouidi E. (2013). Indirect Estimation of VO_2max_ in Athletes by ACSM’s Equation: Valid or Not?. Hippokratia.

[19] Van Erp T. (2019). The Development of Women’s Professional Cycling. J. Sci. Cycl..

[20] Malek M.H., Berger D.E., Housh T.J., Coburn J.W., Beck T.W. (2004). Validity of VO_2max_ Equations for Aerobically Trained Males and Females. Med. Sci. Sports Exerc..

[21] Storer T.W., Davis J.A., Caiozzo V.J. (1990). Accurate Prediction of VO_2_(Max) in Cycle Ergometry. Med. Sci. Sports Exerc..

